# Acoustic levitation combined with laboratory-based small-angle X-ray scattering (SAXS) to probe changes in crystallinity and molecular organisation

**DOI:** 10.1039/d4ra01418a

**Published:** 2024-05-30

**Authors:** Adam Milsom, Adam M. Squires, Jack Macklin, Paul Wady, Christian Pfrang

**Affiliations:** a School of Geography, Earth and Environmental Sciences, University of Birmingham Edgbaston B15 2TT Birmingham UK c.pfrang@bham.ac.uk; b Department of Chemistry, University of Bath South Building, Soldier Down Ln, Claverton Down BA2 7AX Bath UK; c Diamond Light Source, Diamond House Harwell Science and Innovation Campus OX11 0DE Didcot UK; d Department of Meteorology, University of Reading Whiteknights, Earley Gate RG6 6BB Reading UK

## Abstract

Single particle levitation techniques allow us to probe samples in a contactless way, negating the effect that surfaces could have on processes such as crystallisation and phase transitions. Small-angle X-ray scattering (SAXS) is a common method characterising the nanoscale order in aggregates such as colloidal, crystalline and liquid crystalline systems. Here, we present a laboratory-based small-angle X-ray scattering (SAXS) setup combined with acoustic levitation. The capability of this technique is highlighted and compared with synchrotron-based levitation-SAXS and X-ray diffraction. We were able to follow the deliquescence and crystallisation of sucrose, a commonly used compound for the study of viscous atmospheric aerosols. The observed increased rate of the deliquescence–crystallisation transitions on repeated cycling could suggest the formation of a glassy sucrose phase. We also followed a reversible phase transition in an oleic acid-based lyotropic liquid crystal system under controlled humidity changes. Our results demonstrate that the coupling of acoustic levitation with an offline SAXS instrument is feasible, and that the time resolution and data quality are sufficient to draw physically meaningful conclusions. There is a wide range of potential applications including topics such as atmospheric aerosol chemistry, materials science, crystallisation and aerosol spray drying.

## Abstract 2

Single particle levitation techniques allow us to probe samples in a contactless way, negating the effect that surfaces could have on processes such as crystallisation and phase transitions.

## Graphical abstract

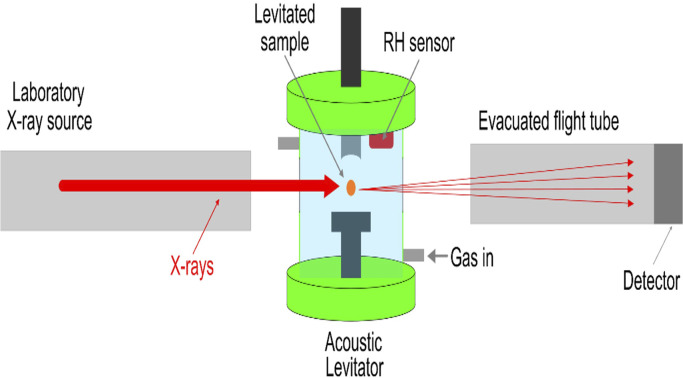

## Introduction

The exploration of nanoscale structures and their properties using small-angle X-ray scattering (SAXS) is a significant area of interest across various scientific disciplines including materials science,^1^ biology,^2^ electrochemistry^3^ and atmospheric chemistry.^4–10^ Acoustic levitation is a non-contact manipulation technique and has emerged as a powerful tool for studying particles on the micrometre-to-millimetre scale, facilitating investigations into their physical and chemical properties.^11,12^ Combined with laboratory based SAXS, acoustic levitation offers a way to probe the structural properties of levitated samples.

Acoustic levitation involves using ultrasonic sound waves to create standing pressure waves in air.^12^ When the frequency and amplitude of these sound waves are controlled, they generate regions of high-pressure (nodes) where particles experience a restoring force and are levitated against gravity. Acoustic levitation enables the isolation and manipulation of microscopic samples, free from contact with solid surfaces, which could introduce undesired effects and alter the properties of the studied materials.

The most common form of acoustic levitator is a single-axis acoustic levitator, although there are many other types which have been elegantly summarised by Andrade *et al.*^13^ The most powerful single-axis levitators are resonant structures made from Langevin horns, where the distance between two transducers or a transducer and a reflector must be precisely controlled to match an integer of the wavelength. As such they are very sensitive to temperature and morphology of the particle, transducer and reflector, but have been used to levitate densities of up to 22.6 g cm^−3^.^14–16^ Due to the complexity in manufacturing and maintaining Langevin horn set-ups, increasingly phased arrays of transducers are being used, first developed as the TinyLev by Marzo *et al.*^17^ This form of levitator uses many small, often cheap transducers in conjunction and carefully controls the phase of the signal applied to each of these in order to produce a very precisely tailored acoustic field. This method has been used to rotationally lock particles,^18^ perform contactless fabrication^19^ and even create graphical displays.^20^

X-ray scattering techniques give information about the repeated order in a sample. A beam of collimated X-rays is directed at a sample and, since the length scale of this order is comparable to the wavelength of X-ray radiation, diffraction of the X-rays occurs. The pattern that arises from this scattering gives information on the nanoscale structures present in the sample. The characteristic spacing between identical scattering planes (*d*) is a function of the momentum transfer (*q*) (eqn (1)).1
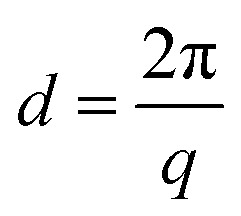
where *q* is a function of the scattering angle (*θ*) and X-ray wavelength (*λ*):2
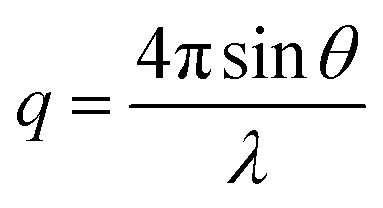


X-ray scattering can be separated into two broad categories – Small- and Wide-Angle X-ray Scattering (SAXS and WAXS). Samples such as self-assembling lyotropic surfactant phases, polymers, porous materials and proteins showing structure and periodicity on the nanometre size scale (probed with SAXS), an order of magnitude larger the Angstrom scale seen in small-molecule crystals (probed with WAXS). In this paper, both SAXS and WAXS are considered.

Combining acoustic levitation with laboratory based SAXS opens up new possibilities for investigating the nanoscale structures of levitated samples. By positioning the levitated particles within the X-ray beam path, it becomes feasible to acquire SAXS data from them. This experimental setup offers the ability to study samples *in situ*, preserving their native properties.

An application of the acoustic levitation experiment coupled with SAXS is the study of atmospheric aerosols.^4,7–9^ Atmospheric aerosols play a crucial role in the climate, air quality, and human health.^21^ Understanding their physical and chemical properties, including their size, morphology, and internal structure, is essential for accurately assessing their impacts on the environment. However, studying aerosols is challenging due to their dynamic nature and diverse compositions. By employing single particle levitation techniques such as optical tweezers,^22,23^ electrodynamic balance^24,25^ and acoustic levitation,^4,7–9,26^ researchers can isolate individual aerosol particles in a controlled environment, avoiding surface interactions and gravitational settling. Coupling acoustic levitation with SAXS enables in-depth characterisation of aerosol structures, providing insights into their internal organisation and interactions with surrounding species. We have previously demonstrated the utility of this experiment in the atmospheric aerosol context with synchrotron-SAXS combined with acoustic levitation.^4,7–9^

Acoustic levitation experiment coupled with X-ray diffraction and SAXS has, to our knowledge, only been carried out at synchrotron facilities. There, the combination of techniques has been used in the observation of crystallization processes,^27^ protein agglomeration^28^ and the investigation of aerosol spray drying.^29^ By precisely controlling the environmental conditions and monitoring the scattering patterns with SAXS, it becomes possible to study the formation and growth of crystals in real-time. This approach provides valuable insights into crystal structures, polymorphism, and phase transitions.

In this paper, we present the experimental design and results of an acoustic levitation experiment coupled with laboratory based SAXS to probe the nanoscale structures of levitated samples. We provide use cases involving sugar crystallisation and dissolution; and changes in the lyotropic liquid crystal (LLC) phases observed in an atmospheric aerosol proxy undergoing humidity changes. The results from this work form a basis for others to apply this method to their own field of study.

## Methods

An acoustic levitator (Tec5, Oberursel, Germany) was employed with a custom-made environmental chamber with inlets and outlets for gas flows and a humidity sensor. The transducer was of a fixed frequency (100 kHz) and a variable power (0.65–5 W) was used to levitate samples. The transducer–reflector distance was between 20–30 mm, adjusted to maintain droplet stability. X-ray transparent mica windows allowed the X-ray beam to pass through, hit the sample and be detected on the other side.

The oleic acid-based LLC phase system was prepared in the following way. Oleic acid (technical grade, 90%, Merck), sodium oleate (Merck) and fructose (Merck) were weighed as a 1 : 1 : 1 wt. mixture and dissolved in methanol with gentle heating to ∼40 °C to make a 10 wt% solution. This solution was used to inject the sample into the levitator.

**Fig. 1:**
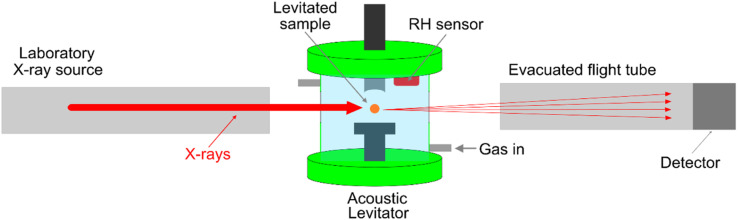
A schematic illustration of the acoustic levitator coupled with a laboratory X-ray source.

Samples were delivered to the levitator *via* injection of a solution, in the case of LLC phase forming mixture, or direct placement of a particle of sucrose into a pressure node of the acoustic levitator (Fig. 1).

Sucrose (Merck) was used as supplied. Both solid crystals of sucrose and saturated aqueous solutions were levitated into a pressure node of the acoustic levitator for the Xeuss experiments. Sucrose dissolved as an aqueous solution from a sachet of sugar for tea and coffee was used for the Malvern Panalytical Empyrean experiments.

Humidity inside the chamber used for the Xeuss experiments was controlled using a Raspberry Pi connected to a DC air pump, which pumped air either through a water bubbler, thus saturating the air with water, or bypassing the water bubbler for humid and dry conditions, respectively. The humidity was monitored inside the chamber *via* a DHT22 temperature and humidity sensor (RH accuracy 2–5%, quoted by the manufacturer) connected to an Arduino, which was connected to the Raspberry Pi. The Raspberry Pi compared the measured RH with the user-set target RH and switched on either the wet pump, if the measured RH was below the target RH, or the dry pump, if the measured RH was above the target.

SAXS data was acquired using the Xeuss 3.0 laboratory SAXS instrument at Diamond Light Source, using an Excillium MetalJet liquid Gallium source to produce exceptionally bright laboratory X-rays at 9.25 keV, and a DECTRIS Eiger M1 detector. Camera lengths of 900 mm and 333 mm were confirmed using a silver behenate calibration. Beam collimation was set to 0.4 mm × 0.4 mm, providing an average count rate of 3.6 × 10^6^ ph s^−1^. The in-air set up was used, providing a large volume for the levitator, whilst minimising the path length through air.

A Malvern Panalytical Empyrean diffractometer was used with a PIXcel3D-Medipix3 1 × 1 detector to measure diffraction from a sugar–water mixture. This was combined with an acoustic levitator sample stage developed in-house, based on the TinyLev developed by Marzo *et al.*^17^ A 160% scaled up version of the TinyLev was used, known as the BigLev, in order to maximise the lifting force and the angles accessible for the X-ray detector. This was operated at 40 kHz and the voltage supplied to the transducers was in the range of 24–32 V, with a power of *ca.* 10 W. This voltage was kept to the minimum that was provide stable levitation to minimise deformation of the droplet from spherical.

A saturated sucrose solution was prepared by dissolving sachets of sugar in distilled water. This solution was then introduced into the levitator using a syringe. The voltage supplied to the levitator transducers was then minimised to reduce the movement of the levitated droplet and to help it retain as spherical shape. It took around 10 minutes before pattern collection to centre the X-ray beam on the droplet by translating the sample stage.

The diffractometer used a Cu anode with a characteristic wavelength of 1.54 Å and a distance to sample of 179.6 mm. Data were collected across the angular range of 2*θ* = 10.20°–40.19°, chosen to cover the most prominent sucrose diffraction peaks.

Humidity was changed and monitored using a small air pump and humidity sensor (DHT22, 2% precision) connected to Raspberry Pi and Arduino microcontrollers. Air was either pumped through a water bubbler for humidification or bypassed the bubbler for dehumidification to room humidity. The humidity change experiments presented were either ramps up to 95 ± 2% relative humidity (RH) or down to room humidity which was *ca.* 55 ± 2% RH.

## Results and discussion

### Cycles of deliquescence and crystallisation in sucrose crystals

Sucrose was chosen to investigate deliquescence and crystallisation. Sucrose is commonly used as a proxy for viscous aerosols due to its presence in atmospheric particles and its well-defined relationship of water diffusivity with water content.^22,30^ Moreover, sucrose is known to form glassy phases under certain conditions, making it a suitable candidate for exploring the transformation between amorphous solids and crystalline phases.^30^ By investigating sucrose deliquescence and crystallisation, we can gain a deeper understanding of the physicochemical properties of aerosol particles and their role in atmospheric processes.

**Fig. 2:**
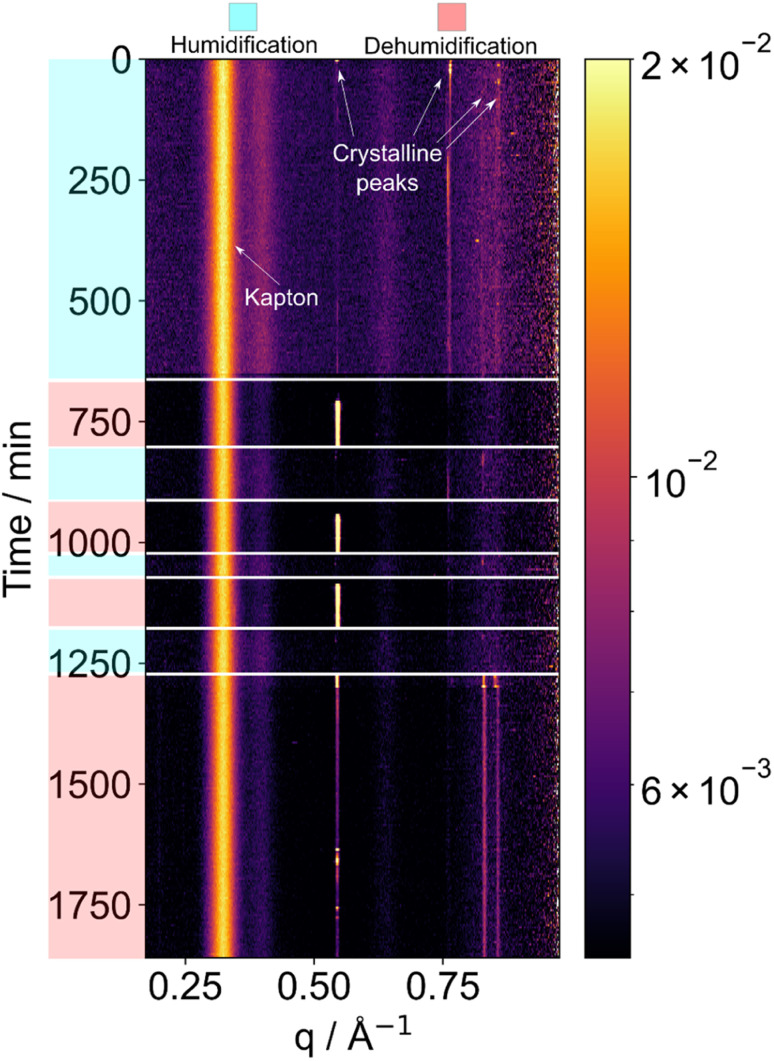
SAXS patterns collected from a levitated particle of sucrose undergoing humidity changes. Humidification is to 95 ± 2% RH; dehumidification is to *ca.* 55 ± 2% RH. Crystalline peaks disappear and reappear upon humidification and dehumidification, respectively.

The time-series of X-ray diffraction patterns are shown as heat map images in Fig. 2 and subsequent figures. Each horizontal strip represents a single diffraction pattern taken at a certain time point (and relative humidity), with bright regions corresponding to Bragg peaks. Increasing scattering vector *q* relates to increasing scattering angle. Bright vertical lines demonstrate persisting Bragg peaks, which show the presence of a crystalline feature. The positions of the peaks are characteristic of sucrose crystals.

We observed deliquescence in sucrose crystals when subjected to an increase in humidity to 95 ± 2% RH (Fig. 2). We allowed the droplet to equilibrate before decreasing the humidity to room humidity (*ca.* 55 ± 2% RH), to induce crystallisation. The crystalline phase returned after dehumidification.

Subsequent humidification–dehumidification cycles resulted in phase changes that were more rapid compared to the initial cycle. This accelerated transformation suggests the possible formation of a glassy phase, wherein the rapid crystalline-to-non-crystalline phase changes may occur predominantly at the surface of the levitated particle after the initial deliquescence event. We suggest that this is due to the very low water diffusion coefficient in glassy sucrose, which is in the order of *ca*. 10^−16^–10^−11^ m^2^ s^−1^.^22,30^ This restricted diffusion would limit the rate of phase change in the bulk of the particle in the timescale of our experiments, meaning that only the surface layers of the particle would lose or gain enough water to realise a phase change. This phenomenon highlights the influence of surface effects on the overall structural evolution of the levitated sucrose particles and that the kinetics of water uptake and loss may need to be considered when predicting cloud droplet formation from viscous aerosols.

**Fig. 3:**
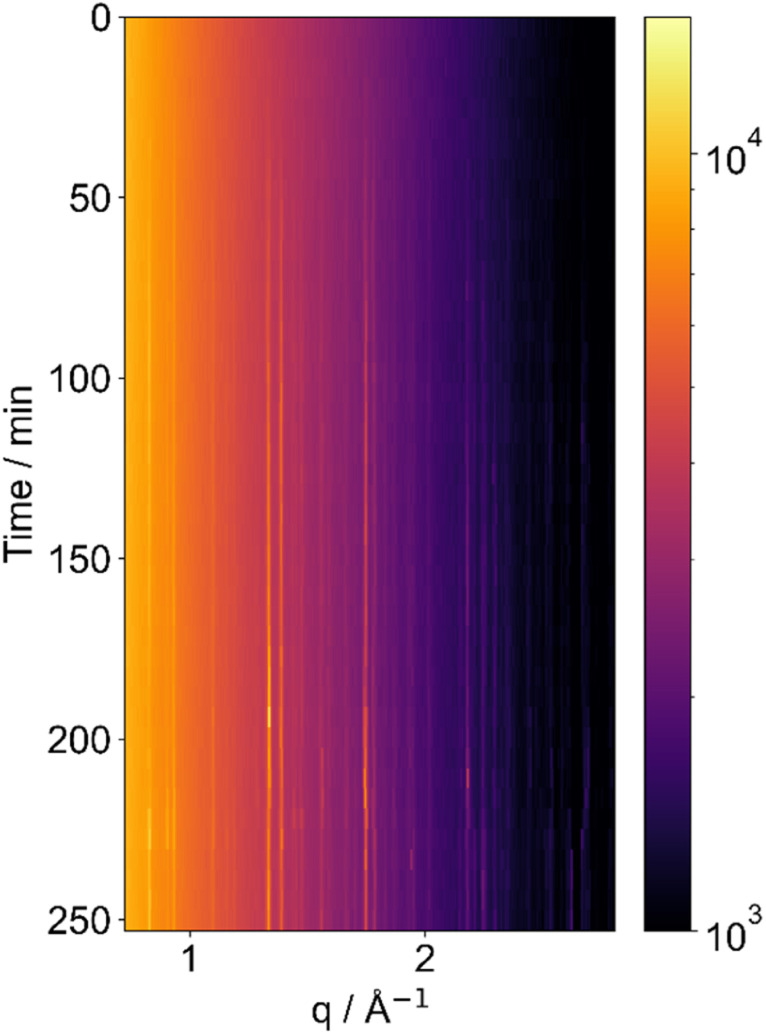
An X-ray diffraction experiment on a levitated aqueous solution of sucrose crystallising over time, demonstrating clear diffraction peaks for crystalline sucrose. This figure shows a series of X-ray patterns collected over time. The intensity of the colour corresponds to the intensity of the X-ray scattering, thus Bragg peaks are shown by colours brighter than the surrounding heatmap.

It is also possible to follow sucrose crystallisation from a levitated aqueous solution. A laboratory-based diffractometer was coupled with an acoustic levitator to achieve this. This diffractometer provided us with a larger *q* range to capture more crystalline peaks than the setup on the Xeuss 3.0 instrument which is really designed more for SAXS experiments. The ability to capture crystalline peaks out to the wider angle is important for identifying crystalline phases of inorganic compounds and small crystals. We again obtained data on sucrose, investigating the crystallisation (Fig. 3). The figure shows a series of peaks that emerge with time, after 40–50 minutes into the experiment, demonstrating that sucrose has crystallised.

There is quite a lot of variation in the intensities of the peaks during the later stages of the experiment. This likely is due to positional instability of the droplet in the X-ray beam as it moves in the acoustic trap. As the droplet moves in and out of the beam, the scattering will become more or less strong as differing amounts of material are illuminated. Additionally, for highly oriented samples, rotation of a crystalline sample will expose different crystal facets to the beam, strongly affecting the intensities of specific diffraction peaks. However, the most important feature for identifying an ordered or crystalline material from X-ray scattering is the set of angles at which the scattering occurs, and therefore the Bragg peak positions which give the crystal symmetry and lattice parameter. This does not change as the droplet moves and as such this method is viable for the identification of compounds or lyotropic phases. In our experiments, the influence of the sound waves on the particles especially in terms of changes in shape and size were minimised by reducing the levitator power.

Synchrotron X-ray diffraction has been used to study crystallisation of different materials from acoustically levitated droplets, including sodium chloride^31^ and aspirin.^32^ However, the reverse process of deliquescence, when crystals dissolve in increased relative humidity as in Fig. 2, has to our knowledge not been previously reported.

### Changes in LLC phases with humidity changes

We chose the oleic acid–sodium oleate–fructose mixture in our study as a cooking and a marine aerosol emission proxy.^33–35^ This mixture has the ability to form different lyotropic liquid crystalline (LLC) phases when exposed to varying amounts of water.^7^ By studying the LLC phase changes of this mixture in response to humidity, we aim to gain an understanding of the dynamic behaviour and potential structural transformations of organic aerosols under changing environmental conditions.

**Fig. 4:**
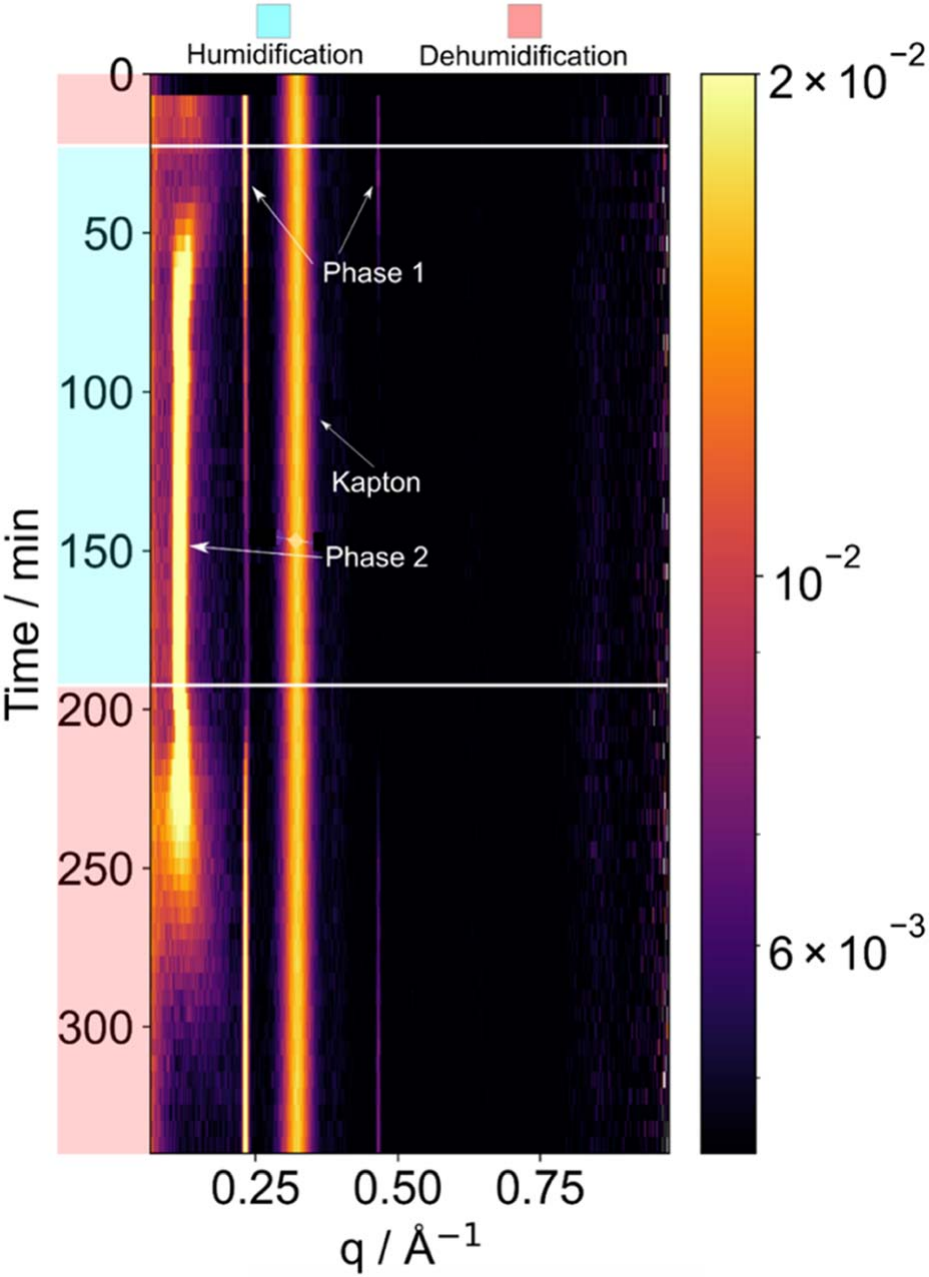
SAXS patterns collected from a levitated droplet of oleic acid–sodium oleate–fructose (1 : 1 : 1 wt.) undergoing humidity changes. Humidification is to 95 ± 2% RH; dehumidification is to *ca.* 55 ± 2% RH. A reversible phase change occurs during a humidification–dehumidification cycle.

We observed peaks consistent with a lamellar phase which took up a significant amount of water during humidification (Fig. 4). The *d*-spacing between repeating lamellar bi-layers increased from 2.7 to 5.5 nm after humidification and equilibration at 95 ± 5% RH. This was reversible upon dehumidification (Fig. 4). The reversible nature of this water uptake highlights the dynamic response of this organic aerosol system to changes in environmental conditions, such as humidity. Understanding and characterising such reversible phase changes in organic aerosols with changes in environmental conditions allow us to understand their behaviour and properties, with implications for atmospheric processes such as cloud droplet formation.^36^

SAXS patterns were collected with a 5 min time resolution, which was a sufficient collection time to identify the lamellar phase peaks and shifts in peak positions. Synchrotron SAXS experiments can produce SAXS patterns in fractions of a second due to the much higher intensity X-ray source.^37^ The combination of synchrotron SAXS with acoustic levitation has previously been explored, where, similarly to this paper, nanomaterial self-assembly was controlled through the RH in an acoustic levitator.^9^ Time and spatially resolved experiments are also made possible with acoustic levitation at synchrotrons by exploiting the smaller beam size and higher photon intensities, where researchers have studied the different effects of RH and ozone concentration at different positions in a particle.^4^ Synchrotron based acoustic levitation experiments have also revealed that there is a wide range of different nanostructures available to atmospheric aerosol proxy by using beam sizes as low of 14 μm FWHM and exposure times of 1 s.^7^ However, synchrotron facilities are very difficult to access, with experiments being very intensive, time-consuming and costly. The difficulty and competition to access facilities also makes it hard to undertake repeat measurements and exhaustive studies. By showing that not all experiments need the high time resolution, we hope to make X-ray scattering from acoustically levitated samples a more widespread technique. Indeed, for the droplet of viscous LLC phase mixture presented here, a 5 min collection time is sufficient to resolve the changes in the SAXS pattern (Fig. 4).

The combination of X-ray scattering with acoustic levitation is a robust and repeatable technique. All of the measurements presented in this paper were successfully repeated multiple times, with changes between samples taking less than a few minutes. The potential duration of data collection is effectively limitless as levitation can continue indefinitely once a sample has been stably loaded into the trap. Very small particles (<100 μm) are difficult to stabilise in the trap and are often lost, although increasing the frequency of the sound waves helps to mitigate this effect. There is positional instability in the trap, with the levitated samples moving around the node which makes it difficult to obtain spatially resolved data on the sample. This effect is diminished for larger particles, higher frequencies of levitation and by eliminating any airflow in the system.

## Conclusions

This study demonstrated the feasibility of the offline levitation-small-angle X-ray scattering (SAXS) setup for investigating nanoscale structures in levitated samples. By combining acoustic levitation with laboratory based SAXS, we have achieved manipulation technique that allows for the isolation and characterization of samples, *in situ* and free from the influence of solid surfaces. Our findings highlight the capabilities of the offline levitation-SAXS setup in probing the structural properties of sucrose crystals and lyotropic liquid crystalline phases – the example systems presented here.

In addition to demonstrating the feasibility of the offline levitation-SAXS setup, our investigation into the exposure of sucrose particles to humidity changes has provided valuable physical insights. The observed increased rate of the deliquescence–crystallisation transitions on repeated cycling could suggest the formation of a glassy sucrose phase. This observation suggests the presence of surface effects and non-equilibrium processes during the deliquescence and crystallisation of sucrose. Crystalline-to-non-crystalline phase changes may occur predominantly at the surface of the levitated particles, particularly after the initial deliquescence event. These findings shed light on the dynamics and stability of crystal structures, as well as the intricate interactions between water molecules and the solid matrix. These physical insights pave the way for further investigations into the role of non-equilibrium effects in water uptake and their implications for the properties of levitated particles.

Overall, the integration of acoustic levitation and laboratory-based SAXS offers a powerful combination of techniques for studying nanoscale structures in levitated samples, providing invaluable insights into their physical and chemical properties without the need to apply for synchrotron SAXS beamtime, which is time restricted and does not allow for long-term (*i.e.* days-to-weeks long) experiments on levitated samples. This technique could be applied to other laboratory-based SAXS instruments provided there is a large enough sample chamber to accommodate an acoustic levitator. By advancing our understanding of these complex materials, this research opens up opportunities for numerous applications in fields such as materials science, biotechnology, and pharmaceutical research.

## Conflicts of interest

There are no conflicts to declare.

## Supplementary Material
